# Summary from the NCI clinical trials planning meeting on next generation of clinical trials in non-muscle invasive bladder cancer^*^

**DOI:** 10.1177/23523735251319185

**Published:** 2025-02-24

**Authors:** Andrea Apolo, Brian C Baumann, Hikmat Al-Ahmadie, Leslie Ballas, Rick Bangs, Kenneth Brothers, Stephanie Cooper Greenberg, Scott Delacroix, James J Dignam, Jason A Efstathiou, Adam S Feldman, Jared C Foster, Noah M Hahn, Emma Hall, Donna E Hansel, Jean Hoffman-Censits, Ashish M Kamat, Sophia C Kamran, Francesca Khani, Seth P Lerner, Robert Lipman, Bhupinder Mann, David McConkey, James McKiernan, Tracy L Rose, Angela B Smith, Catherine Tangen, Abdul Tawab Amiri, Chana Weinstock, Pamela J. West, Matthew I Milowsky, Peter C Black

**Affiliations:** 1 Genitourinary Malignancies Branch, Center for Cancer Research, National Cancer Institute, National Institutes of Health, Bethesda, MD, USA; 2 Department of Radiation Oncology, Springfield Clinic, Springfield, IL, USA; 3 Department of Radiation Oncology, University of Pennsylvania, Philadelphia, PA, USA; 4 Department of Pathology and Laboratory Medicine, Genitourinary Pathology Service, Memorial Sloan Kettering Cancer Center, New York, NY, USA; 5 Department of Radiation Oncology, Cedars Sinai Medical Center, Los Angeles, CA, USA; 6 Patient Advocate, Bladder Cancer Advocacy Network, Bethesda, MD, USA; 7 Patient Advocate, National Cancer Institute Bladder Cancer Task Force, Rockville, MD, USA; 8 Patient Advocate, Johns Hopkins Greenberg Bladder Cancer Institute, Baltimore, MD, USA; 9 Department of Urology, Louisiana State University Health Science Center, New Orleans, New Orleans, LA, USA; 10 Department of Public Health Sciences, University of Chicago, Chicago, IL, USA; 11 Department of Radiation Oncology, Massachusetts General Hospital, Harvard Medical School, Boston, MA, USA; 12 Department of Urology, Massachusetts General Hospital, Harvard Medical School, Boston, MA, USA; 13 Division of Cancer Treatment and Diagnosis, National Cancer Institute, NIH, Bethesda, MD, USA; 14 Departments of Oncology and Urology, Johns Hopkins University, Greenberg Bladder Cancer Institute, Baltimore, MD, USA; 15 Clinical Trials and Statistics Unit, The Institute of Cancer Research, London, UK; 16 Division of Pathology and Laboratory Medicine, University of Texas MD Anderson Cancer Center, Houston, TX, USA; 17 Department of Urology, University of Texas MD Anderson Cancer Center, Houston, TX, USA; 18 Department of Pathology and Laboratory Medicine, Weill Cornell Medicine, New York, NY, USA; 19 Scott Department of Urology, Dan L. Duncan Cancer Center, Baylor College of Medicine, Houston, TX, USA; 20 Cancer Therapy Evaluation Program, National Cancer Institute, NIH, Rockville, MD, USA; 21 Herbert Irving Comprehensive Cancer Center, Columbia University, New York, NY, USA, Department of Urology, Columbia University, New York, NY, USA; 22 Lineberger Comprehensive Cancer Center, University of North Carolina, Chapel Hill, NC, USA; 23 Department of Urology, Lineberger Comprehensive Cancer Center, University of North Carolina, Chapel Hill, NC, USA; 24 SWOG Statistical Center, Fred Hutchinson Cancer Research Center, Seattle, WA, USA; 25 Coordinating Center for Clinical Trials, National Cancer Institute, NIH, Bethesda, MD, USA; 26 Center for Drug Evaluation and Research, U. S. Food and Drug Administration, Silver Spring, MD, USA; 27 Emmes Company, Rockville, MD, USA; 28 Department of Urologic Sciences, University of British Columbia, Vancouver, BC, Canada

**Keywords:** non-muscle invasive bladder cancer, BCG, clinical trial, radiation therapy

## Abstract

The National Cancer Institute organized a virtual Clinical Trials Planning Meeting (CTPM) on ‘Defining the next generation of clinical trials with combination therapies in non-muscle invasive bladder cancer (NMIBC)’ led by the Bladder Cancer Task Force of the NCI Genitourinary Cancers Steering Committee. The purpose of this meeting was to accelerate advances in clinical trials for patients with high-risk NMIBC. The meeting delivered a multidisciplinary expert consensus on optimal strategies for next-generation clinical trial designs in NMIBC with prioritization of combination therapies. Two clinical trial concepts were developed for potential implementation within the National Clinical Trials Network.

## Introduction and overview of objectives

The National Cancer Institute organized a virtual Clinical Trials Planning Meeting (CTPM) on ‘Defining the next generation of clinical trials with combination therapies in non-muscle invasive bladder cancer’ led by the Bladder Cancer Task Force of the NCI Genitourinary Cancers Steering Committee. This CTPM occurred on December 8–9, 2022, and was chaired by Drs. Peter Black, Andrea Apolo, Brian Baumann and Matthew Milowsky. The purpose of this meeting was to accelerate further advances in clinical trials for patients with high-risk non-muscle invasive bladder cancer (NMIBC). The meeting focused on the evaluation of strategies incorporating intravesical therapy, systemic therapy, radiation therapy, and combination therapies toward a goal of improving bladder preservation rates and survival outcomes for patients with high risk NMIBC, including Bacille Calmette-Guérin (BCG)-unresponsive NMIBC.

The objectives of this effort in NMIBC were: (i) to establish consensus on key components of trial design; (ii) to explore the feasibility of a multi-arm adaptive randomized clinical trial (RCT); (iii) to develop two clinical trial concepts focused on bladder preservation for patients with high risk NMIBC for implementation through the NCTN, and (iv) to plan biomarker integration into NMIBC clinical trials.

Potential deliverables of this CTPM include determination of a multidisciplinary expert consensus on optimal strategies for next-generation clinical trial designs in NMIBC, prioritization of combination therapies for NMIBC, determination of feasibility of adaptive multi-arm RCT in NMIBC, identification of biomarkers to incorporate into trials (with the aim of validating candidate predictive biomarkers for each treatment arm) and recommendations for tissue handling for the most robust and impactful downstream analyses in these trials. Streamlining the design of the 2 clinical trial concepts proposed in the context of this CTPM for potential implementation within the NCTN was among key deliverables of this enterprise.

## Clinical trial challenges in NMIBC

Clinicians, statisticians, scientists, and patient advocates convened in this CTPM to focus their efforts on finding multidisciplinary consensus in clinical trial design and the study of correlative biomarkers in clinical trials for patients with NMIBC. This necessitated concise presentations on the state-of-the-art with respect to clinical management of patients with NMIBC, clinical trial methodology, the science behind candidate biomarkers and methods of specimen collection and processing in clinical trials.

### Defining NMIBC disease states

The management of high-risk NMIBC poses a formidable challenge, necessitating the development of novel and efficacious therapeutic approaches. To attain this objective and optimize patient outcomes, it is imperative to establish meticulously designed clinical trials. These trials should encompass carefully selected endpoints, eligibility criteria, evaluations, statistical analyses, and correlative studies. Standardizing trial design and endpoints offers advantages by facilitating the generation of more comparable and robust datasets for meta-analyses and for cross-trial clinical interpretation and application of data. Importantly, the context-specific endpoints would be meaningless if the initial characterization of the NMIBC disease state is inaccurate.

High-risk NMIBC denotes a subset of NMIBC that carries an elevated risk of progressing to muscle-invasive bladder cancer (MIBC) or resulting in mortality. For the purpose of designing clinical trials, it is recommended to consider all high-grade (HG) tumors (Ta, Tis/ carcinoma in situ (CIS) and T1) regardless of size, as high-risk NMIBC.^1^ While some guidelines propose using tumor size as a discriminating factor (e.g., considering some TaHG tumors < 3 cm in size as intermediate risk), size estimation is subject to substantial variability and operator dependence, consequently leading to potential misinterpretation of trial outcomes.

High-risk NMIBC can be further subdivided based on previous exposure to intravesical BCG therapy: BCG-naïve (no prior intravesical BCG administration), BCG-exposed (prior BCG within 24 months but not meeting BCG-unresponsive criteria), and BCG-unresponsive (according to the United States Food and Drug Administration [US FDA] guidance).^2,3^ BCG-unresponsive high-risk NMIBC encompasses high-grade Ta/T1 recurrence within 6 months or CIS within 12 months of adequate BCG therapy. BCG-exposed NMIBC encompasses BCG resistance (presence of high-grade Ta or CIS at the 3-month evaluation after induction BCG) and delayed relapse that does not meet the criteria for BCG-unresponsive disease. For the purpose of these sub-classifications, adequate BCG therapy is defined as a minimum of 5 out of 6 doses of induction BCG and 2 doses of maintenance or second induction BCG in patients with recurrent/persistent high-grade Ta/CIS, and 5 out of 6 doses of induction BCG in patients with recurrent/persistent T1 tumors.

The different disease states within high-risk NMIBC are associated with distinct clinical implications. For example, BCG-unresponsive high-risk NMIBC is associated with a higher risk of progressing to MIBC or death compared to BCG-naïve high-risk NMIBC.

### Patient perspectives: unmet needs in NMIBC care

The unmet needs in NMIBC care from the patient perspective focused on three broad categories: quality of patient-centered data, quality of tests and treatments, and quality of life. A survey investigated levels of tolerance to treatment side-effects, respondent-ranked clinical relevance of various trial efficacy measures, and differences in responses between patients, caregivers, and healthcare provider.^4^ In some cases, caregivers and providers expressed higher acceptance rates of treatment-related toxicities than patients did. Clinician perceptions are not a substitute for patient preferences.

Quality of data requires strong evidence that treatments are well-tolerated from the patient perspective. Formal patient surveys are needed before, during, and after clinical trial treatment. Quality of life measures need to be captured prospectively as an endpoint in clinical trials. There needs to be validated questions to measure the patient experience with treatment and their quality of life with the goal of developing a database of patient experiences that can be employed across many clinical trials.

The quality of tests and treatments needs to evaluate the positives and negatives of intravesical, intravenous, and oral treatment including the frequency of administration and associated toxicity. Although bladder preservation is a key goal, the treatment to preserve the bladder should not be perceived as worse than a radical cystectomy. While risk-benefit is part of quality-of-life measures, it should also be extended to include costs, i.e., lost wages, impact on employment, travel for appointments, child/elder care, long-term psychological effects, and costs related to fear and anxiety as well as others. The GU Cancer Steering Committee Summary of Disease-Specific Priorities states to “incorporate measures to evaluate patient QOL in clinical trials of patients with NMIBC, MIBC, and metastatic bladder cancer and/or other urinary tract malignancies.” Let us put into action the need to evaluate QOL in patients at all stages of bladder cancer in clinical trials.

### Financial toxicity: implications of treatment intensification for patients with NMIBC

Financial toxicity (FT), which refers to the negative financial impact of a cancer diagnosis on households, has been linked to various detrimental outcomes such as declines in health-related quality of life (HRQOL), reduced treatment options, and increased mortality rates.^5^ Therefore, when developing new treatments for conditions like NMIBC, it is crucial to consider the implications of FT.

FT often drives treatment decisions and compliance with prescribed treatment, significantly influences outcomes, and plays a central role in survivorship. NMIBC in particular incurs substantial direct and indirect costs due to FT, as indicated by limited cross-sectional survey-based data. For instance, a study by Casilla-Lennon et al. revealed that 25% of patients with NMIBC experienced financial toxicity, which impacted their treatment decisions and led to delays in care.^6^ Another study by Ehlers et al. found that FT was not associated with the stage or type of treatment but rather with the time elapsed since treatment.^7^ Surprisingly, 63% of patients expressed a preference to discuss the cost of treatment regardless of their FT status. Moreover, a cross-sectional study by Bhanvadia and colleagues demonstrated that patients diagnosed with bladder cancer within the past year had higher odds of feeling a lack of control over their current financial situation compared to those diagnosed over a year ago.^8^

A systematic review on FT in urologic malignancies called for the use of validated instruments to consistently assess FT, particularly for comparisons of new treatments.^9^ Any treatment intensification strategy should incorporate the consideration of FT while weighing the cost trade-offs associated with recurrence, TURBTs, missed work for treatment, and the upfront expenses of ancillary tests like molecular testing and newer therapies that can potentially reduce recurrence.

### Defining a role for trimodal therapy in T1 bladder cancer

High-grade non-muscle invasive bladder cancer (NMIBC) is a challenging entity to treat, particularly if it is recurrent after BCG. Radical cystectomy is the standard treatment for patients with BCG-unresponsive NMIBC^10^ and in the highest risk cases without prior intravesical therapy.^11^ However, many patients decline to undergo radical cystectomy due to the known associated morbidity and mortality as well as negative consequences for quality of life such as impaired sexual function and altered body image.^12,13^ In addition, many patients are not eligible for radical cystectomy because of their performance status or comorbidities. Several second-line drug treatments have been evaluated to manage recurrent high-grade NMIBC, and three have been approved by the FDA for patients with BCG-unresponsive CIS with or without concurrent Ta/T1 tumors.^14–18^ Nonetheless, a significant proportion of patients will still recur after these novel therapies and there remains a need to improve upon the clinical outcomes in this patient population.

Concurrent chemoradiotherapy following maximal TURBT, also known as trimodal therapy, is a safe and effective treatment option for patients with MIBC. It is associated with low toxicity rates, excellent functional outcomes and good quality-of-life in comparison to radical cystectomy.^19,20^ A recent multi-institutional propensity score matched and weighted study including 722 patients with MIBC (440 who underwent radical cystectomy, 282 who underwent trimodal therapy) found similar oncologic outcomes between the two treatments with respect to cancer-specific and disease-free survival.^21^ The question then is whether we can translate this successful approach to the recurrent high-grade NMIBC disease space.

Radiation has been previously explored as a treatment option for NMIBC, with mixed results. For example, a randomized trial of radiation therapy alone versus conservative treatment (intravesical BCG or observation) for high-grade T1 tumors did not find any advantage with the use of radiation.^22^ However, a separate study involving combined chemoradiation therapy after a maximal TURBT at the University of Erlangen demonstrated promising results, with a complete response rate of 88% in a cohort of 141 patients. Disease-specific survival rates were 82% and 73% at 5- and 10-years respectively. Of surviving patients, more than 80% retained their bladder.^23^

This and other experiences provided the impetus for the prospective cooperative group clinical trial, NRG/RTOG-0926.^24^ In this single-arm, phase II trial, patients with high-grade recurrent T1 bladder cancer received 61.2 Gy concurrently with either cisplatin or mitomycin/5-fluorouracil after maximal TURBT.^24^ Of 34 evaluable patients with a median follow-up of 4.2 years (>5 years in surviving patients), the three-year freedom from cystectomy rate was 88%. The three- and five-year overall survival rates were 69% and 56% respectively. The cumulative incidence of distant metastasis at three- and five-years was 12% and 19%, respectively. Eighteen patients had grade 3 adverse events, mostly hematologic, and one grade 4 neutropenia. These early results demonstrate that trimodal therapy may be an effective and well-tolerated option for select patients with high-grade T1 disease who have recurred despite intravesical therapy.

Moving forward, combining radiation with therapeutics such as immunotherapy or antibody-drug conjugate therapies may further improve clinical outcomes for patients with high-grade T1 NMIBC. Combining chemoradiation with an immune checkpoint inhibitor is currently being explored for MIBC in the ongoing NRG/SWOG 1806 (NCT03775265) and KEYNOTE-992 (NCT04241185) trials. There is evidence that radiation may activate the immune response, resulting in antitumor activity.^25,26^ Furthermore, this response may be augmented by combining radiation therapy with immune checkpoint inhibitors.^27–29^ Therefore, administration of radiation or chemoradiation with a therapeutic such as an immune checkpoint inhibitor may contribute to improved long-term clinical responses in patients with NMIBC.

Trimodal therapy has a role for high-grade recurrent, BCG-unresponsive T1 bladder cancer, which may be augmented with the addition of novel therapeutics to improve local control and long-term clinical outcomes, allowing patients to retain their native bladder. Future opportunities include the use of promising biomarkers to identify appropriate patients for consideration of this approach.

### Pathology considerations for determining endpoints in NMIBC trials

Clinical trial endpoints, as well as entry criteria, may be confounded by interobserver variability and suboptimal standardization in pathologic interpretation of bladder specimens. The two main areas in which this is most likely to impact NMIBC clinical trials are in the substaging of T1 tumors and in the diagnosis of urothelial carcinoma *in situ* (CIS). The challenge with T1 tumors is that these cancers show a range of invasion into the lamina propria, including cases with minimal invasion below the basement membrane to cases with an extensive amount of invasion either in single or multiple foci. The most recent version of the World Health Organization Classification of Urinary and Male Genital Tumors (2022), the American Joint Committee on Cancer (AJCC) 8^th^ edition, the Genitourinary Pathology Society (GUPS) and the International Society of Urological Pathology (ISUP) all recommend that pathologists perform T1 substaging due to its prognostic importance, but there has been no consensus on the optimal method for this, despite several methods having been proposed.^30–32^ Both GUPS and ISUP specify particular acceptable methods for estimating extent of lamina propria invasion, such as providing the histoanatomic depth (e.g., above or below the muscularis mucosae/vascular plexus, providing depth/diameter of invasion factoring in single vs multiple foci), but there is a lack of prospective validation and adequate data to promote the optimal method.^33,34^ Importantly, not all methods can be consistently applied due to the fragmented nature of transurethral resection specimens and lack of surface orientation in many cases. Standardization of a substaging methods for patients in clinical trials may become an important tool for assessing risk of disease progression but additional work is needed.

The diagnosis of urothelial CIS, which is particularly important for both entry criteria and endpoints in NMIBC trials, represents a second critical pathology consideration. In particular, if histologically apparent CIS is present in the vicinity of a high grade papillary urothelial carcinoma, some pathologists consider this as part of the papillary tumor (i.e., a “shoulder lesion”) and do not mention CIS separately in the pathology report. The extent of CIS that should be present in order to warrant mention of it separately from a Ta tumor has not been established. Further complicating the issue is that transurethral resection (TUR) specimens often have multiple fragments of tissue, and therefore the linear “length” of urothelial CIS and its relationship to a concurrent papillary tumor are often difficult to determine. Additionally, there is known interobserver variability and numerous pitfalls in the diagnosis of CIS, including reactive epithelial changes, changes due to intravesical chemotherapy such as mitomycin, and radiation-related changes.^35,36^ To ameliorate these potentially confounding issues in NMIBC trials, central review of the pathology specimens for enrolled patients by designated subspecialized genitourinary pathologists and clear-cut thresholds to diagnose CIS in the context of a papillary lesion would optimize more standardized interpretation. This approach will also help in reducing interobserver variability in grading papillary urothelial carcinoma. In the modern era, this is becoming more feasible due to an increase in digital slide scanning capabilities within more pathology departments, since digital slide images can be shared more readily than original glass slides.

### Regulatory considerations in NMIBC trials

Regulatory approval of a therapy for a given indication requires the demonstration of safety and efficacy through adequate and well-controlled investigation(s). It is therefore imperative that trials designed to support approval of a therapeutic product in a given indication be designed in a manner that ensures adequacy, including precision around the methods of analysis and any applicable statistical methods (Code of Federal Regulations 314.126).^37^ Demonstration of sound evidence of effectiveness becomes a crucial component of the US FDA's benefit-risk assessment of a new therapy or indication. Thus, per statutory requirement, precision in trial design is crucial, as is ensuring accuracy of outcome measurement and minimizing bias when approaching trials in NMIBC.

NMIBC has historically been a disease space with an unmet clinical need for therapeutic drug development, with relatively few therapies approved by the FDA. Additionally, global BCG supply shortages have become a persistent issue, heightening the need to develop alternatives as safe and effective therapies for treatment of NMIBC. Recognizing the unmet clinical need in this disease space, and cognizant of the need to standardize trial design elements to ensure homogeneity and interpretability of trials, the FDA joined with the academic bladder cancer community to address issues related to trial design in NMIBC. Workshops designed to address trial design in NMIBC included an FDA/American Urological Association Bladder Cancer Workshop held on May 6, 2013 and an FDA Public Workshop held on November 18 and 19, 2021. These workshops resulted in several publications^38–41^ and an FDA guidance related to trial design in BCG-unresponsive NMIBC finalized in 2018.^3^ A draft update of this guidance document is currently under public review.^42^ Among other objectives, these efforts aimed to standardize definitions of disease states and definition of prior therapy that would be required prior to calling a patient's disease BCG-unresponsive. These efforts also provided discussion around accuracy in definition of clinically relevant endpoints to ensure clinical trial interpretability and success. Some of the takeaways of these workshops are further referenced in the next section of this manuscript.

The three recent FDA approvals of novel therapies in BCG-unresponsive CIS (pembrolizumab,^15^ nadofaragene firadenovec,^43^ and nogapendekin alfa inbakicept^18^ have been based on single-arm data in patients with CIS at trial entry, a path to regulatory approval previously discussed in the 2018 FDA guidance. Results of very similar trials testing TAR-200 (with and without the immune checkpoint inhibitor cetrelimab)^44^ and cretostimogene grenadenorepvec (with^45^ and without^46^ pembrolizumab) have been reported. FDA had stated that patients with BCG-unresponsive CIS could potentially be studied in either a single-arm trial or in a randomized controlled trial (RCT).^3^ A single-arm design was considered appropriate for a clinical setting where a RCT was considered unethical or infeasible and because the complete response rate would be negligible in the absence of therapy. Historically, the standard of care for patients with BCG-unresponsive CIS was radical cystectomy, a procedure associated with morbidity, mortality and a negative impact on quality of life and without a guarantee of long-term cure. However, as more therapies are FDA-approved and more widely used (including off-label use of intravesical chemotherapy), the necessity for and feasibility of an RCT to support approval is becoming a pertinent consideration.

The 2021 FDA workshop and subsequent 2022 publication therefore included discussion around potential design issues for RCTs in the BCG-unresponsive setting, including considerations around the appropriate choice of control arm. At the time of these discussions, it was still unclear whether current practice patterns were such that the FDA-approved therapies for BCG-unresponsive NMIBC could be deemed “standard of care.” Thus, randomizing patients to an active FDA-approved control arm was still thought to be potentially challenging. Importantly, while the control arm of an RCT must be considered an accepted U.S. standard of care, the control arm is not required to be an FDA-approved therapy (e.g., off-label intravesical chemotherapy may potentially be used). Ultimately, as the therapeutic options in this disease space evolve, the use of a randomized control trial design in this setting is considered feasible and is increasingly encouraged.

For patients without evaluable disease (i.e., patients with papillary disease resected at the time of trial entry), a time-to-event endpoint (e.g., recurrence-free survival) is needed to demonstrate efficacy, however time-to-event endpoints cannot be interpreted based on results of a single-arm trial. Therefore, a randomized controlled trial design is necessary to demonstrate efficacy for this population.^3,41^

Sponsors developing novel therapeutics in NMIBC are encouraged to meet with the FDA to discuss issues related to dose optimization and trial design early in development. Optimal trial designs will always be context dependent, and for trials intending to support FDA submission, meeting with the appropriate FDA review division to discuss the specifics of trial design and objectives is strongly recommended. We note that while FDA representatives were invited to the CTPM meeting under discussion to provide a regulatory background and as observers, no input was provided on the clinical trial concepts discussed and presented in this manuscript. FDA does not endorse specific therapeutic approaches discussed in this manuscript that are not FDA-approved, and clarifies that financial considerations are not accounted for in FDA regulatory decisions.

### Clinical trial endpoints in NMIBC

In February 2019 the FDA published final guidance for developing drugs and biologics for treatment of BCG-unresponsive NMIBC. This disease state is defined above in “Defining NMIBC Disease States.” The guidance laid out a straightforward registration pathway design based on the principle that randomizing patients with BCG-unresponsive disease to a minimally effective drug as a concurrent control raised ethical concerns. Because effective drugs were not available and the alternative treatment is cystectomy, single-arm trials of patients with BCG-unresponsive CIS with or without papillary disease are appropriate. The primary endpoint should be complete response (CR) rate and durability of response in patients with CIS. The FDA requires that adequate durability of CR is an important component of efficacy. Future trials should consider combination therapy and randomized trials can stratify by CIS +/-Ta/T1 or Ta/T1 only.

CR requires cystoscopy showing no visible papillary tumors and cytology showing no high-grade cells. Biopsies should be performed for cause for visible papillary disease and at a defined endpoint which can be 3, 6 months or end of study (e.g., 12 months). Patients undergoing for cause biopsy must have a negative biopsy or low-grade cancer with negative cytology in order to remain on trial. For intravesical therapies, a second primary tumor of the upper tract or prostatic urethra with negative bladder biopsies may not count towards the primary endpoint, but the suitability for these patients to remain on trial treatment will have to be decided on an individual basis. For systemic therapies used for NMIBC, a second urothelial primary tumor outside the bladder is not consistent with CR and patients must come off trial.

Investigational treatment should not be discontinued for persistent CIS at 3 months unless T1HG cancer is also present. Re-induction in this scenario has proven efficacious in three trials.^18,44,46^ In patients with CIS, biopsies to adjudicate CR are recommended but not required. The expert urologic oncology community recommends this as the sensitivity of urine cytology, while relatively high, is not enough to definitively rule out persistent or recurrent CIS.

Event-free survival (EFS) allows inclusion of all patients with CIS +/- Ta or T1 cancer and patients with Ta or T1 high grade cancer without CIS. This may be a secondary endpoint in a single arm trial and may contribute additional evidence of efficacy. An event for EFS is defined as the first occurrence of the following: histologically proven high-grade bladder cancer (e.g., persistent or recurrent CIS at pre-specified time point usually within 3–6 months), high-grade upper tract urothelial carcinoma (for systemic therapies only), high-grade urothelial carcinoma of the prostatic urethra, MIBC, clinical evidence of metastatic disease, or death due to any cause (adapted from SWOG S1605).^17^

For patients with MIBC treated with radiation, bladder intact disease-free survival (BIDFS) is a novel endpoint and may be a useful secondary endpoint in BCG-unresponsive NMIBC, although this endpoint has not been used for FDA approval of any products to date. This is a composite endpoint including histologically proven recurrence of MIBC, clinical evidence of nodal or metastatic disease, radical cystectomy, or death due to any cause. Freedom from cystectomy is an important secondary endpoint as cystectomy is still considered standard of care in this disease state.

## NMIBC clinical trial concepts and design considerations

The CTPM included 3 working groups who joined their expertise to address scientific and clinical challenges in the NMIBC clinical trials field. Two working groups focused on one clinical trial concept each and a third working group focused on the biomarker studies ancillary to the trials. The stage was set for the working groups by a discussion of innovative trial designs and statistical considerations.

### Applying innovative trial designs to NMIBC

With the accelerated speed of innovative technology advances both in diagnostics and therapeutics, novel trial designs which facilitate rapid accrual and incorporation of new agents or approaches during the conduct of the study are in tremendous need. The multi-arm, multi-stage (MAMS) clinical trial design facilitates such flexibility and the pre-planned ability to bring in additional study arms not initially envisioned at a date after the initial study activation.^47,48^ In a MAMS design, an initial stage(s) designed to rapidly close underperforming arms is utilized followed by enrollment to an additional stage for arms successfully exceeding predefined statistical metrics in the initial stage(s). Examples of early metrics may include safety as assessed by treatment related toxicity rates, tumor response rate, or early event-free survival benchmarks. In contrast, later stage metrics may include more definitive endpoints such as full event-free survival targets or overall survival. An example of the MAMS stages is presented in Table 1. By evaluating multiple different treatment arms within the context of a singular, yet flexible trial, the MAMS approach allows information of the relative value of each arm to be assessed more rapidly than would a series of separately conducted clinical trials.

**Table 1.** Multi-Arm Multi-Stage (MAMS) design stages/segments.

Stage	Primary Outcome	Secondary Outcomes
Pilot	Toxicity	Response Rate
		Event-Free Survival
		Overall Survival
Activity Stage 1	Response Rate	Toxicity
		Event-Free Survival
		Overall Survival
Activity Stage 2	Event-Free Survival	Toxicity
		Response Rate
		Overall Survival
Efficacy Stage 3	Overall Survival	Toxicity
		Response Rate
		Event-Free Survival

**Figure 1.**
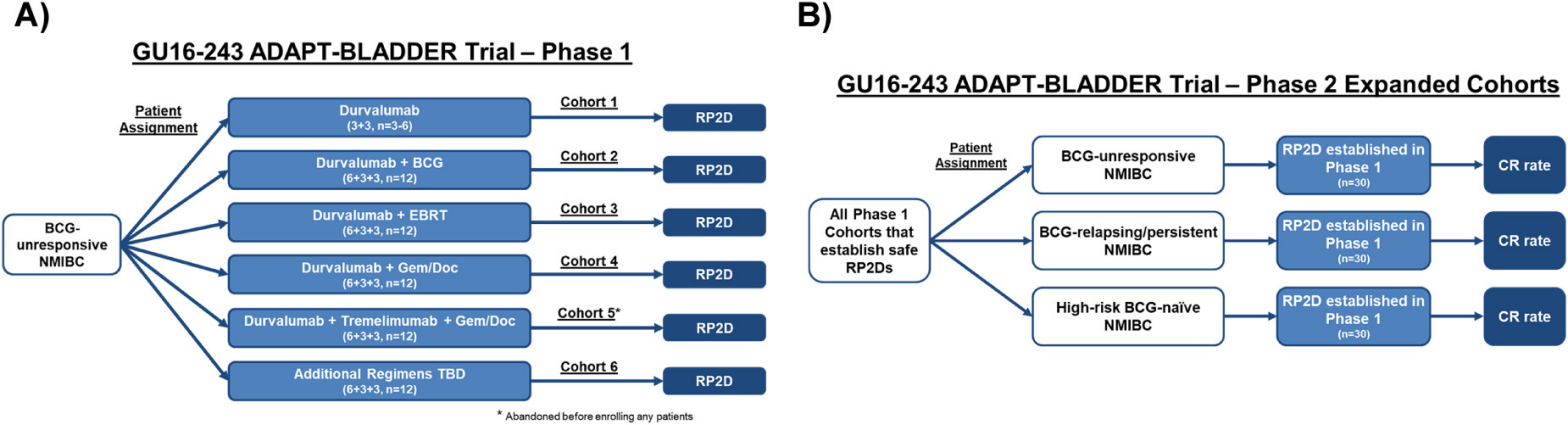
HCRN GU16-243 ADAPT-BLADDER trial design. A) Phase 1 Design. B) Phase 2 Design.

Inspired by the first NMIBC CTPM held in 2015,^49^ the HCRN GU16-243 ADAPT-BLADDER trial was one of the first studies in NMIBC to utilize a MAMS design to evaluate novel combination therapy regimens. The trial initially aimed to study the safety of three regimens in BCG-unresponsive NMIBC patients: Cohort 1 – durvalumab monotherapy; Cohort 2 – durvalumab + intravesical BCG; and Cohort 3 – durvalumab + 3 fractions of immunogenic-intent EBRT. The safety of each combination regimen was tested in at least twelve phase 1 patients in a 6 + 3 + 3 enrollment design. Regimens clearing phase 1 safety evaluation could seamlessly move forward to phase 2 efficacy assessment of complete response rate in CIS patients (Figures 1A-1B). Phase 1 safety and complete response results were recently published for Cohorts 1–3.^50^ True to its intent, the study was adapted after initial activation to add Cohort 4 – durvalumab + intravesical gemcitabine + intravesical docetaxel (Gem/Doc) and Cohort 5 – durvalumab + tremelimumab + intravesical Gem/Doc. Cohort 5 was abandoned prior to activation, however, cohort 4 enrollment is ongoing. The trial infrastructure allows for further cohorts of new combination regimens to be added in the future. Through the use of a MAMS design, the ADAPT-BLADDER trial has evaluated the phase 1 safety and preliminary efficacy of three separate combination therapy regimens in a shorter time frame than would be possible through the conduct of three independent phase 1 clinical trials. While not suitable for all situations, the experience with the ADAPT-BLADDER trial demonstrates the feasibility of conducting MAMS trials in NMIBC populations and presents attractive advantages to traditional drug development paradigms.

### Statistical considerations

In designing cancer clinical trials, the aim is usually to identify safe and promising treatments or treatment combinations, determine whether such treatments are active in impacting outcomes related to efficacy, toxicity or quality of life and ultimately generate the evidence to effect a change in clinical practice. Multi-stage designs combine these aims, utilising intermediate endpoints for decision making at interim assessment points and multi-arm approaches allow for simultaneous evaluation of a number of experimental treatments often with planned adaption to drop (or add) arms as data emerge.^51^ There can be logistical complexity with both approaches but compared to the historical approach of sequential 2-arm phase II and phase III trials, the statistical efficiency can lead to more rapid curtailment of unpromising or ineffective treatments and/or more rapid implementation of effective new treatments.

In the phase II setting, randomisation between multiple experimental arms will minimise bias and a randomised control group will allow direct comparison or “benchmarking” to support decision making based on the magnitude of an observed treatment effect albeit in a “signal finding” capacity often with relaxed type 1 error rates.^52,53^ Multi-arm multi-stage designs (randomized or non-randomized) were considered for both trial concepts discussed at the CTPM and further development will need to consider whether adaption at the interim assessment stage is based around dropping arms for futility or lack of activity, retaining all arms that meet pre-defined threshold activity levels or selecting from all arms, or from those that meet activity thresholds, the most promising arm(s) for later stage comparative evaluation (“pick-the-winner”). Bayesian approaches can give rise to decision making based on probabilities of treatment futility or the predictive probability of success in a future phase III trial^54^ and may also be appropriate in this setting.

The estimand framework encourages a precise description of the clinical question of interest reflecting the trial objective, specified in terms of: treatment, population, endpoint, intercurrent events and population-level summary measure.^55^ In the NMIBC setting, careful consideration of the strategy for handling intercurrent events, i.e., events that occur after treatment initiation that affect the ability to measure and/or interpret the endpoint of interest, is particularly relevant. Examples of such events might be low grade NMIBC recurrence or events relating to treatment discontinuation – a known issue with BCG maintenance therapy.

Key to the design of any trial is the choice of endpoint. Endpoints need to be robust and reproducible and in phase III the primary endpoint needs to have direct relevance to the patients not least so that results can then impact clinical practice. In a multi-stage design or where interim stop/go decisions are planned, short term intermediate endpoints that do not require prolonged follow-up will maximise the opportunity for statistical efficiency. In NMIBC trials, overall survival, and in lower risk disease even progression-free survival, is likely an infeasible outcome measure due to the relatively good prognosis of patients. Here a degree of pragmatism is required in selecting endpoints that occur with sufficient frequency to lead to feasible sample sizes. Stakeholder engagement in defining appropriate and meaningful outcome measures is essential to maximise the chances of successfully delivering a trial.

### Feasibility of novel trial designs within NCTN infrastructure

During the roundtable discussion, the feasibility of novel trial designs within the National Clinical Trials Network (NCTN) was discussed including the importance of having a nimble process for real time data analysis, the adding and dropping of study arms, negotiating and accessing novel agents from different pharmaceutical sponsors, as well as a facile process for correlative sample collection and central pathology review. Dr Bhupinder Mann, CTEP Head Genitourinary Therapeutics discussed the feasibility of working with different sponsors and cited successful examples of NCTN studies involving multiple companies such as the PAPMET trial in papillary renal cell carcinoma.^56^ To ensure an efficient review process within the steering committee, the importance of a close collaboration between the genitourinary steering committee co-chairs and its members and the bladder cancer task force co-chairs and its members was emphasized. With limited resources within the NCTN and the importance of ensuring that studies will complete accrual, it will be difficult to accomplish both the adding and dropping of arms over the course of years and at the same time putting the best arms forward as phase 3 trials. The roundtable concluded with a recognition of the importance of finding the best clinical question, determining feasibility, and matching the clinical question with the study objectives and the most appropriate trial design acknowledging that there will always be compromises.

## Clinical trial concepts and recommendations

### SWOG trial concept in exposed and unresponsive phase 2/3 randomized combination trial

Working group 1 discussed the first concept: ‘A multi-arm RCT testing multiple combination therapies in BCG-unresponsive NMIBC’ with a focus on the design and feasibility of a multi-arm, adaptive, randomized controlled trial testing combination therapies. This trial would prioritize a patient-centric approach to assessing the burden of treatment in NMIBC. The group discussed the possibility of an adaptive trial but determined that a simpler design would be required to make the trial feasible within the NCTN.

The proposed trial would be a randomized Phase II/III trial with two or more treatment arms in patients with BCG-unresponsive carcinoma in situ (CIS). No definitive recommendations were made for specific treatments in each arm. There was some consensus that the most appropriate control arm would be sequential intravesical gemcitabine and docetaxel, which has evolved as the most commonly used regimen in usual practice in the United States, although it has not been tested formally in a clinical trial. It was acknowledged that the control arm does not have to be FDA approved therapy. The other treatment arms would prioritize combination therapies, including novel agents in combination with gemcitabine/docetaxel.^15,18,43,57^ Systemic immunotherapy could be incorporated into combination therapies, although some participants favored restricting the treatment arms to intravesical therapies to avoid having systemic therapy in one or more arms but not in others.

The primary endpoint of the Phase II component would be complete response at 6 months. This time point would allow for re-induction therapy as needed in patients with persistent Ta/CIS at the 3-month evaluation. This phase of the trial would include a futility analysis based on CR rates at 6 months to enable an ineffective regimen to be dropped early. If this futility threshold is surpassed, the treatment would continue until the target sample size of patients with BCG-unresponsive CIS is treated. Arms that meet the phase II criteria for success would continue accrual in the phase III portion of the trial. Factors used to determine an arm's success may include durability of response at 12 months (after treatment start), drug safety profiles, patient-reported outcomes and treatment costs. As this concept will be refined, the investigators will identify detailed criteria for a trial arm's success or elimination.

The FDA has previously stated that a randomized trial in this disease state should assess CIS ± Ta/T1 separately from Ta/T1 without CIS. In this case the primary endpoint of the CIS arm would be CR at 6 months and the primary endpoint of the Ta/T1 arm would be event-free survival at a specified time point (e.g., 18 months). The panel suggested the alternative strategy of treating CIS and Ta/T1 together with a single endpoint of EFS at 18 months. Randomization would be stratified according to presence/absence of CIS.

The panel explored potential sample size for an EFS endpoint in a mixed patient population. Based on reported outcomes for nogapendekin alfa inbakicept in the Quilt 3.032 trial and assumptions of patient cohort composition, the EFS at 18 months in the control group in phase 3 would be estimated to be 42%. A regimen would be considered superior to the “standard arm” if the EFS at 18 months were 54%. This translates into a hazard ratio of 1.41. Using a two-sample test of proportions (e.g., a Z test), such a between-arm difference could be detected with 80% statistical power using a one-sided alpha = 0.05 with a total sample size of 213 per arm. Given that the precise phase II success criteria have not yet been finalized, it is difficult to determine how many arms would be carried forward to the phase III portion of this trial. Thus, these calculations are offered to ensure the feasibility of such a trial design, as further modifications are likely as the trial concept matures.

### NRG trial concept in high-grade T1 disease

Working group 2 focused on the second clinical trial concept: ‘A multi-arm RCT testing trimodal therapy versus alternative therapies in high risk T1 bladder cancer’. The objective of this trial is to test whether an immune checkpoint inhibitor improves oncologic outcomes when added to chemoradiation and whether a combination of immune checkpoint inhibitor and a novel intravesical chemotherapy delivery system, TAR-200, can achieve outcomes superior to those with chemoradiation. The study would enroll patients with either recurrent T1 high-grade urothelial carcinoma after prior intravesical therapy or de novo T1 with very-high-risk features who otherwise would be treated with cystectomy as standard of care. Patients would have to have had a complete TURBT and would need to be eligible for radiotherapy, chemotherapy, and immunotherapy. The randomized phase II design would enroll patients into one of three arms including concurrent chemotherapy and radiotherapy; concurrent chemotherapy, radiotherapy and checkpoint inhibitor; or TAR-200 and checkpoint inhibitor. The group further discussed some alternative designs with respect to inclusion criteria, treatment arms, feasibility and whether a two-arm trial would be able to achieve the goals if a three-arm trial proved infeasible.

Based on feedback from the CTPM, the NRG trial concept was amended to two-arm phase II trial randomizing patients with high-grade T1 disease eligible for cystectomy by AUA/SUO criteria to receive definitive chemoradiation vs. definitive radiation plus 1 year of concurrent and adjuvant pembrolizumab. This trial was approved by the NCI's GU Steering Committee and is slated to begin enrollment in late 2024/early 2025 as NRG GU 014.

### Biomarkers

Working group 3 focused on the study of biomarkers in NMIBC trials and exploring strategies and methods to facilitate the design of standard processes for tissue, blood and urine collection, as well as maximizing their impact.^58^ The group concentrated their efforts to identify areas of consensus on methods of handling, storage and banking of biospecimens from all potentially relevant sources for future studies including, omics, ctDNA and utDNA analyses, artificial intelligence/deep learning studies and the banking of imaging data for radiomic studies. Use of fresh, frozen, and fixed tissue, as well as blood and urine processing was discussed. The consensus of these recommendations was developed to outline the discussed methods and to guide incorporation into clinical trials.^59^

## Summary

A number of substantive and collaborative ideas were generated as direct outcomes of this CTPM. The two trial concepts (summarized in Table 2) are being further developed within the NCTN and next steps to implementing the trials have been taken. The NRG trial is scheduled to begin enrollment in early 2025. A guidance document has been generated to guide correlative biomarker studies in these and other future NMIBC trials.^59^ The multidisciplinary and collaborative approach of the NCI CTPM highlighted challenges and strategies that may be broadly applied to successfully execute the next generation of clinical trials in NMIBC.

**Table 2.** Summary of clinical trial concepts discussed at clinical trials planning meeting.

	Concept 1	Concept 2
NMIBC context	BCG-Unresponsive NMIBC	Trimodal therapy for T1
Patient inclusion	Defined by FDA guidance CIS +/- Ta/T1 and Ta/T1 without CIS	T1 requiring definitive local therapy (primary or recurrent; with or without prior therapy)
Patient exclusion	-	Prior pelvic radiation
	-	Extensive CIS
Study design	Randomized controlled trial	Randomized controlled trial
Control arm therapy	Gemcitabine-docetaxel (Intravesical) or FDA-approved agent	Trimodal therapy alone
Experimental arm therapy	Novel agent alone or in combination with control or third agent	Trimodal therapy plus additional agent
		Alternative non-radiation-based therapy (e.g., TAR-200)
Stratification	Presence/absence of CIS	Prior intravesical therapy
Primary endpoint	Option 1: Separate analysis of CIS +/- Ta/T1 (CR @ 6 months) and Ta/T1 without CIS (EFS @ 18 months)	Bladder intact event-free survival @ 3 years
	Option 2: Combined analysis of all comers: EFS @ 18 months stratified by presence/absence of CIS	
Secondary endpoints	Duration of response (in patients with CIS)	
	Progression to MIBC	Progression to MIBC
	Cystectomy-free survival	Cystectomy-free survival
	Metastasis-free survival	Metastasis-free survival
	Cancer-specific survival	Cancer-specific survival
	Overall survival	Overall survival
Statistical considerations	Superiority	Superiority
Sample size	TBD	TBD
Patient surveillance	Cystoscopy and cytology every 3 months for 2 years, then every 6 months for additional 3 years	Cystoscopy and cytology every 3 months for 2 years, then every 6 months for additional 3 years
	Recommend re-biopsy at 6 or 12 months	Annual CT abdomen/pelvis
Special considerations	Allow re-induction for Ta/CIS	

Abbreviations: CIS: carcinoma in situ; FDA: Food & Drug Administration; EFS: event-free survival; MIBC: muscle-invasive bladder cancer; TBD: to be determined.
